# Effect of a Web-Based Nutritional and Physical Activity Intervention With Email Support (the EDDY Program) on Primary School Children’s BMI Z-Score During the COVID-19 Pandemic: Intervention Study

**DOI:** 10.2196/50289

**Published:** 2024-09-19

**Authors:** Alina Gansterer, Paula Moliterno, Rhoia Neidenbach, Caroline Ollerieth, Sarah Czernin, Juergen Scharhag, Kurt Widhalm

**Affiliations:** 1 Austrian Academic Institute for Nutrition, Vienna, Austria; 2 Sports Medicine, Exercise Physiology and Prevention, Department of Sport and Human Movement Science, Centre for Sport Science and University Sports, University of Vienna, Vienna, Austria; 3 Department of Medicine III, Division of Gastroenterology and Hepatology, Medical University of Vienna, Vienna, Austria

**Keywords:** childhood obesity, BMI, prevention intervention, physical activity, nutrition, nutritional, school-based, web-based, COVID-19, diet, child, childhood, children, pediatric, pediatrics, weight, obesity, obese, exercise, school, student, students, youth

## Abstract

**Background:**

COVID-19 mitigation measures enhanced increases in children’s weight and BMI due to decreased physical activity and increased energy intake. Overweight and obesity were major worldwide problems before the pandemic, and COVID-19 increased their severity even more. High BMI directly correlates with health disadvantages including cardiovascular diseases, musculoskeletal disorders, and mental health diseases. Therefore, it is vitally important to develop counteracting interventions to maintain children’s health during exceptional situations like pandemics. However, worldwide data from such interventions are limited, and to our knowledge, no suitable study has been carried out during the pandemic in Austria.

**Objective:**

This study was conducted to examine a 15-week web-based intervention with email support, the EDDY (Effect of Sports and Diet Trainings to Prevent Obesity and Secondary Diseases and to Influence Young Children’s Lifestyle) program and the effect of nutritional education and physical activity on children’s BMI z-score during the COVID-19 pandemic in Vienna, Austria.

**Methods:**

The intervention consisted of 3 weekly videos—2 physical activity and 1 nutritional education video, respectively—and a biweekly email newsletter for the parents. This study was conducted in a Viennese primary school from February to June 2021 by a team of physicians, nutritionists, and sports scientists, including both professionals and students. The study population included an intervention group (who received web-based nutritional and physical activity training) and a control group (who received no intervention), comprising in total 125 children aged 8 to 11 years. Due to COVID-19 mitigation measures, the control group was a comparative group observed during the prior school year (2019-2020). Anthropometric measurements were obtained before and after the intervention in both groups.

**Results:**

Due to a high dropout rate (n=57, 45.6%) because of the mitigation measures, there were 41 children in the intervention group and 27 in the control group. At baseline, the BMI z-score was 1.0 (SD 1.1) in the intervention group and 0.6 (SD 1.2) in the control group (*P*=.17). After the study period, the BMI z-score decreased by 0.06 (SD 0.21) in the intervention group, whereas it increased by 0.17 (SD 0.34) in the control group (*P*<.001). Comparing the change in BMI z-scores within BMI categories in the intervention group and control group revealed a statistically significant difference in the normal-weight children (*P*=.006). Further results showed that the decrease in BMI z-score was significant in the intervention group among both boys (*P*=.004) and girls (*P*=.01).

**Conclusions:**

A web-based intervention with combined nutritional education and physical activity training might be an adequate tool to lessen the enhanced increase in body weight during a pandemic. Therefore, additional studies with greater sample sizes and different locations are needed. As the implementation of such intervention programs is essential, further studies need to be established rapidly.

## Introduction

Since the implementation of COVID-19 restriction measures to reduce the transmission of the disease, the daily routines of adults and children have changed dramatically. As a consequence, an increase in energy intake and a decrease in physical activity (PA) has been reported in schoolchildren [1-3], affecting their BMI and increasing the severity of overweight and obesity [1,2]. In Austria, studies have also reported an increase in body weight and BMI during lockdowns [4-6], affecting boys especially. The restriction of daily activities, the stay-at-home orders, and the closure of playgrounds and sports facilities led to decreased levels of PA in children. Moreover, school PA education classes were interrupted, which may have influenced the observed decrease in cardiorespiratory fitness measurements reported previously [4]. A decline in PA was also described worldwide [3,7], being greater among boys and those who lived in apartments or houses with limited space. Being physically active has proven beneficial in improving physical fitness, BMI [8], and markers of cardiovascular health, as well as bone and mental health [9].

In contrast, obesity is known to have many health disadvantages. There is a direct correlation between elevated BMI and elevated risk for cardiovascular diseases, diabetes, musculoskeletal disorders, cancer, and mental disorders [10,11]. Therefore, implementing programs to prevent and tackle childhood obesity is one of the main research aims of the World Health Organization [12] to maintain children’s health.

Further studies suggest that school-based interventions play an essential role in the prevention of childhood obesity [13-16]. The combination of PA and nutritional education is most effective [17]. Additionally, parental involvement in nutrition and PA [3] interventions is crucial to promote healthier lifestyle behavior. Since parenting practices and parent-child interactions are formative in children’s health-related behaviors, studies suggest that interventions with parental involvement are more effective [18]. Moreover, parents and children ask for realistic approaches, such as ideas for active games and activities or healthful meals and snacks that families can enjoy [19].

Furthermore, study authors have recommend the opportunity to use web-based interventions to promote healthy behaviors [14]. Online training programs have been suggested to support healthy eating and active movement [5,20-22]. A parent-focused online intervention in Australia led to improvement in dietary-related practices and self-efficacy [23]. Also, evidence indicates that implementing web-based programs at schools could be beneficial, as a high number of people can be reached and they are easy to conduct [24]. Results from web-based studies with other outcome variables, such as alcohol drinking prevention, tobacco knowledge, and eating disorder prevention in children and adolescents, also indicate the usefulness of web-based interventions in children and adolescents [24-26]. Communication tools provide useful means to develop digital health content that can be accessed easily through social media platforms such as YouTube. However, to be effective, this content should be age tailored, involve limited equipment [20], and be able to motivate children [27]. Compared to face-to-face programs, online intervention programs offer advantages such as convenience and self-engagement with PA. On the other hand, family income could be a limitation regarding internet access, mobile device availability, proper space [28], or adequate equipment [29] to perform PA.

Recent studies have called for further investigations focusing on a combination of settings [15,17] and on parents’ involvement [30]. As the COVID-19 pandemic and its effects on children increased the severity of childhood overweight and obesity [1,2], it is of major importance to find a way to execute a web-based preventative intervention in this specific setting in order to gather more knowledge, as there is expected to be an increased risk of other infectious disease outbreaks with climate change [31].

Therefore, we aimed to examine the effects of a 15-week web-based nutritional and PA intervention with email support and parental involvement on BMI z-score among children aged 8 to 11 years during a school year that took place in the context of the COVID-19 pandemic in Vienna.

## Methods

### Study Design

This intervention study was designed to involve children and parents as part of the EDDY (Effect of Sports and Diet Trainings to Prevent Obesity and Secondary Diseases and to Influence Young Children’s Lifestyle) program. The EDDY program focuses on nutrition and PA training to prevent childhood obesity and takes place in primary schools in Vienna, Austria. It was founded by the Austrian Academic Institute for Nutrition in Vienna in cooperation with the Institute for Sport Science of the University of Vienna by a professional team consisting of physicians, nutritionists, and sports scientists, as well as students studying for these professions. The EDDY program was implemented in 2012 in the primary school VS (Volksschule; German for “primary school”) Haebergasse 1120, Vienna, and is part of the school’s fourth-grade curriculum [32]. Thus, all fourth-grade students are candidates to participate in the study with previous consent from their parents or legal guardians. The planned intervention and all anthropometric measurements were performed in the school setting.

In the 2019-2020 school year, a 6-month school-based nutritional and PA intervention was planned. Due to the outbreak of the COVID-19 pandemic, the school-based intervention had to be stopped after 3 weeks in March 2020, but baseline and end measurements could still be obtained. During the 2020-2021 school year, an on-site intervention was difficult to schedule due to the sustained restriction measures. Therefore, a 15-week web-based nutritional and PA intervention program was developed covering areas of knowledge and practices related to healthy meals, snacks, and drinks, as well as PA. Moreover, parents received an informative email newsletter.

Due to the COVID-19 pandemic, the web-based intervention could only be carried out at one school due to mitigation restrictions and the Austrian government being stringent with school studies during that time. To simplify the intervention’s feasibility, the intervention sample comprised all 3 of the school’s fourth-grade classes.

### Ethical Considerations

The study was approved by the research ethics committee at Sigmund Freud University in Vienna, Austria (PAFGRW9O@EFQV885378). Legal guardians of all children enrolled in the study provided written informed consent for participating in the study after receiving an informative letter about the procedure of the intervention. The children themselves gave their own consent. The ability to opt out was provided at any point. All gathered data in this study were anonymized. As the EDDY program is part of the school curriculum, compensation was not provided to study participants.

### Description of the Intervention

**Figure 1.**
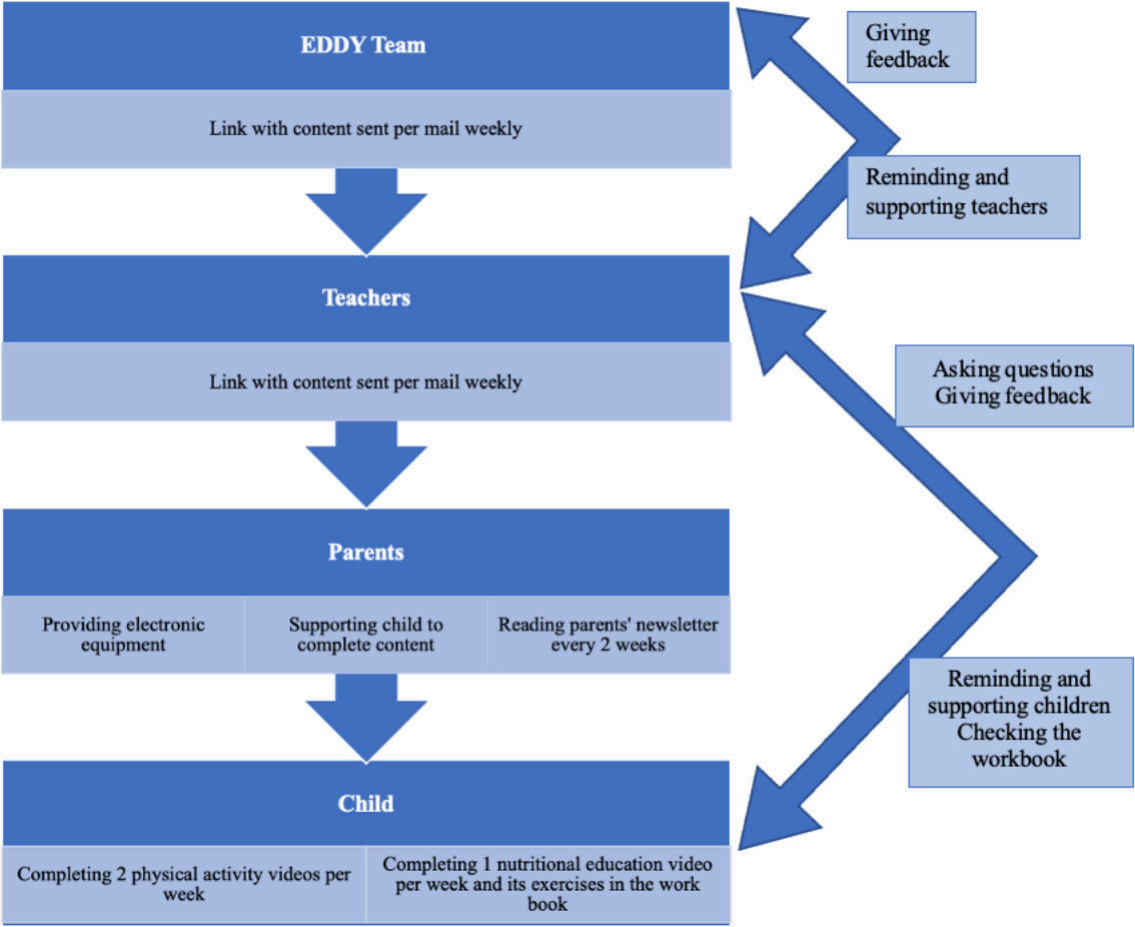
Description of the intervention and each participant’s role. EDDY: Effect of Sports and Diet Trainings to Prevent Obesity and Secondary Diseases and to Influence Young Children’s Lifestyle.

The web-based intervention implemented in this study consisted of 2 main components: (1) nutritional education and (2) stimulating a PA routine during a 15-week period from February to June 2021 with 3 videos per week. Educational and movement content was provided as videos via YouTube. As the videos were not publicly available, the parents received a weekly email with a link to access the content. Therefore, parents needed to instruct their children to watch the videos and support them with electronic equipment. Figure 1 shows a description of the intervention and each participant’s role.

### Nutrition

Previous nutritional lessons from the EDDY program have been adapted for web-based training [32]. The educational content was recorded by nutritional scientists and medical school students. It includes both theoretical and practical age-targeted content focusing on food with high nutrient content (vegetables, fruits, and legumes), relevance of timing in eating, and skills to prepare and select healthier food choices, such as recipes for easy, healthy snacks and identification of suitable portion sizes. The content is based on food groups and the healthy plate [33]. Videos were delivered once a week with a duration ranging from 10 to 20 minutes.

Additionally, a workbook with worksheets was provided, including questions about the content of the videos, quizzes, and practical tasks. Progress was monitored for feedback and positive reinforcement.

### PA Training

The PA training was designed and conducted by a team of sports scientists, physicians, and students. Since we provided web-based videos and did not have face-to-face contact with the children, the workouts were designed to not require any equipment and use only the participants’ own body weight.

Each week, the children received two 25-minute videos via YouTube; one focused on strength training and the other on endurance training. The strength training started with a warm-up phase (coordination exercises and warm-up games) that focused on the correct execution of the subsequent strength exercises. The intensity of the exercises was slow to moderate. The warm-up phase was followed by 10 to 15 minutes of strength training that incorporated 4 to 6 exercises (eg, squats, stretch jumps, push-ups or plank exercises, spider walking, duck walking, sit-ups, and wall sitting) for the entire body. Two series with a repetition count of 15 to 20 repetitions were targeted per exercise. The strength training was taught in a playful way that included the use of children’s stories.

The endurance training was structured as follows: The primary focus was to keep children moving continuously over a 25-minute period. The intervention included standing endurance runs, knee runs, heel runs, jumping jacks, frog jumps, boxing exercises, and gymnastic exercises with moderate to high intensity. In addition, dances were choreographed to child-friendly pop music to increase the entertainment factor. All videos ended with a short relaxation and cool-down session. Here, yoga and stretching movements were incorporated and relaxation stories involving fantasy journeys were told in order to achieve better body awareness and to recover from the PA.

The videos were repeated 2 times during the 15-week intervention period to create confidence and enthusiasm for improving physical fitness through the repetition effect.

### Intervention for Parents

A major part of the intervention was the inclusion of parents or legal guardians. During the intervention period, the parents received an email newsletter every 2 weeks with healthy lifestyle recommendations, nutrition facts, recipes, and tips on how to increase the amount of time spent physically active. Information on how to increase PA during the period of social distancing and restriction measures (eg, self-use of outdoor gyms and playgrounds and indoor family workouts) was a particular focus. Also, parents received all the intervention videos for the children from their teachers (in the teachers’ experience, parents are always more conscientious when being texted by school staff). Therefore, they were in charge of providing electronic equipment and supporting their child in completing the weekly videos.

### Involvement of Teachers

The teachers acted as a link between the children and their legal guardians. At baseline, they received workbooks and distributed them to the children. All videos, newsletters, and information during the 15 weeks of the intervention were sent to the teachers and forwarded to the study participants via email. Teachers interacted closely with the study team, reminded the children to complete the videos, and checked on their workbooks approximately every 2 weeks. Every few weeks, the teachers gave feedback on how the children responded to the content of the intervention.

### Participants

#### Intervention Group

**Figure 2.**
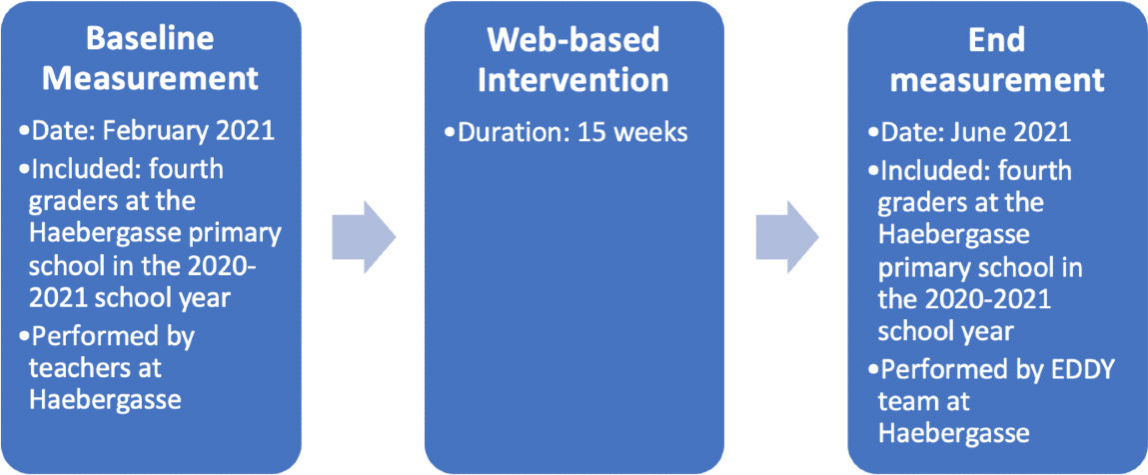
Description of the intervention group's timeline. EDDY: Effect of Sports and Diet Trainings to Prevent Obesity and Secondary Diseases and to Influence Young Children’s Lifestyle.

Primary school children from the fourth grade at VS Haebergasse 1120 in the 2020-2021 school year were included and received 15 weeks of web-based nutritional and PA intervention. Anthropometric measurements were performed in February 2021 (before the intervention) and June 2021 (after the intervention) at the school facility. The children were not randomly allocated to the intervention group; instead, all fourth graders in the 2020-2021 school year were eligible for the study to make it more feasible for teachers to organize. As the EDDY program is part of the school’s fourth grade curriculum, only children opting out did not receive the weekly videos (Figure 2).

#### Control Group

**Figure 3.**
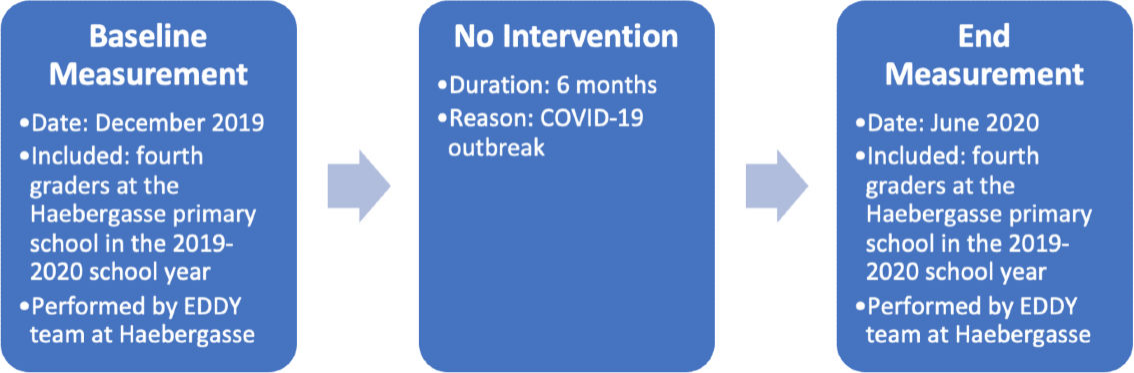
Description of the control group timeline. EDDY: Effect of Sports and Diet Trainings to Prevent Obesity and Secondary Diseases and to Influence Young Children’s Lifestyle.

For this analysis, primary school children from the fourth grade at VS Haebergasse 1120 in the 2019-2020 school year comprised the control group. During this study year, no repetitions were recorded. Therefore, no children were part of both the control and intervention groups. Anthropometric measurements were performed in December 2019 (before the planned intervention) and June 2020 (after the planned intervention) at the school facility. The planned 6-month intervention could not be carried out due to the outbreak of COVID-19 and state-imposed school closures. The children were not randomly allocated to the control group; instead, all fourth graders in the 2019-2020 school year were eligible for the study to make it more feasible for teachers to organize. Therefore, only children opting out were not included in the measurements (Figure 3).

#### Inclusion Criteria

To be eligible for this analysis, the participants needed to fulfill the following eligibility criteria: be aged 8 to 11 years, be a student in the fourth grade at the specific primary school where the intervention took place, complete all measurements, and provide written informed consent (both legal guardians and children).

#### Exclusion Criteria

The exclusion criteria were as follows: be aged <8 or >11 years, not be a student in the fourth grade at the specific primary school where the intervention took place, miss at least one measurement, change class or school during the intervention, and not provide written informed consent (both legal guardians and children).

### Outcome Measures

Body weight was measured to the nearest 0.1 kg with a bioelectrical impedance analysis scale (Tanita MC-780MA) without shoes or excessive clothing. Body height was evaluated to the nearest 0.1 cm with a portable stadiometer (Seca) with the participant standing and maintaining their head in the Frankfort horizontal plane position without shoes.

BMI was calculated as weight (kg) divided by square of height (m^2^) and classified according to age and sex. BMI z-scores were used for further analysis [34]. SDs for the BMI z-scores were calculated and BMI categories were classified in reference to Kromeyer-Hauschild et al [35]. The obesity category was considered as the sum of obesity and extreme obesity.

Measurements were performed at 2 time points for both groups at the primary school. Due to COVID-19–related school closures in December 2020 and January 2021, baseline measurements of the intervention group were postponed to February 2021. Due to ongoing COVID-19 restrictions and social limitations, the EDDY team could not perform baseline measurements of the intervention group themselves; therefore, they were performed by the teachers at school with the school doctor’s equipment. To obtain accurate measures, the EDDY team standardized the measurement procedures among the teachers by giving instructions via video. End measurements of the intervention group were performed by the EDDY team. The control group was measured by the EDDY team at both measurement time points.

### Statistical Analysis

For statistical analysis, all data were analyzed with SPSS (version 27; IBM Corp). Continuous variables were expressed as means (range) and categorial variables were expressed as absolute numbers or proportions. Age and sex differences between excluded and included students were determined with the *χ*^*2*^ test and Mann-Whitney *U* test. Age differences between the study groups were determined using the Mann-Whitney *U* test. Shapiro-Wilk tests were performed to determine standard distribution for BMI, age, weight, height, and BMI z-score at baseline and for BMI z-score differences over the study period. BMI z-score differences in groups and between sex over the study period were assessed using Mann-Whitney *U* tests. For all the above tests, statistical significance was considered as *P*<.05. BMI z-score differences between study groups within the BMI categories were tested with the Mann-Whitney *U* test. Therefore, the Bonferroni test was used as a post hoc analysis, for which statistical significance was considered as *P*<.017.

## Results

### Study Population at Baseline

At baseline, 125 children (age range 9.25 to 11.75 years) were eligible to participate in the study (n=68 boys and n=57 girls); there were 56 children in the intervention group and 69 students in the control group. Eventually, 57 children (45.6%) could not be included due to quarantines or absence from school and missing measurements; 15 children in the intervention group and 42 in the control group. The total number of excluded students included 22 girls and 35 boys. The mean age in the dropout group was 10.34 (SD 0.57; range 9.25‐11.75) years. The excluded and included students did not differ by sex (*P*=.15) but did differ by age (*P*=.02).

Finally, there were 41 children in the intervention group and 27 in the control group. Anthropometric data for the study participants are shown in Table 1.

The mean age in the intervention group was 10.2 (SD 0.5; range 9.41‐11.17) years and 10.0 (SD 0.5; range 9.33‐11.08) years in the control group. There was no significant difference in age between the intervention and control groups (*P=*.09).

At baseline, n=8 (20%) children in the intervention group and n=4 (15%) children in the control group were classified as obese, with excessive BMI (≥90th percentile) for their age and sex. In the intervention group, 2 (1 female, 1 male) of 8 children with obesity were classified as extremely obese (≥99.5th percentile). Additionally, in the control group, 1 (female) of 16 children with normal weight was classified as low weight but was represented as normal weight in the figures and tables due to the small number. BMI classification at baseline testing and end testing in each study group is shown in Table 2.

**Table 1.** Age and anthropometric data of study participants at baseline.

	Intervention group, mean (SD)			Control group, mean (SD)			*P* value
	Total (n=41)	Female (n=23)	Male (n=18)	Total (n=27)	Female (n=12)	Male (n=15)	
Age (years)	10.2 (0.5)	10.0 (0.4)	10.4 (0.6)	10.0 (0.5)	9.9 (0.4)	10.1 (0.6)	.09^a^
Weight (kg)	43.0 (10.8)	40.4 (8.6)	46.3 (12.6)	39.5 (9.6)	39.3 (11.1)	39.7 (8.6)	.24^a^
Height (m)	142.8 (6.4)	142.3 (6.4)	143.5 (6.5)	142.1 (7.3)	142.0 (7.9)	142.2 (7.1)	.69^b^
BMI (kg/m^2^)	21.0 (4.5)	19.8 (3.6)	22.4 (5.2)	19.4 (3.8)	19.3 (4.4)	19.5 (3.4)	.16^a^
BMI z-score	1.0 (1.1)	0.8 (0.9)	1.2 (1.3)	0.6 (1.2)	0.5 (1.4)	0.7 (1.1)	.17^b^

a Mann-Whitney *U* test.

b Student *t* test.

**Table 2.** BMI classification in the study group at baseline testing and end testing.

		Control group participants (n=27), n (%)	Intervention group participants (n=41), n (%)
**Baseline testing**			
	Normal weight	16 (59)	24 (59)
	Overweight	7 (26)	9 (22)
	Obese	4 (15)	8 (20)
**End testing**			
	Normal weight	17 (63)	25 (61)
	Overweight	4 (15)	7 (17)
	Obese	6 (22)	9 (22)

### Change in BMI z-Score After Intervention

**Figure 4.**
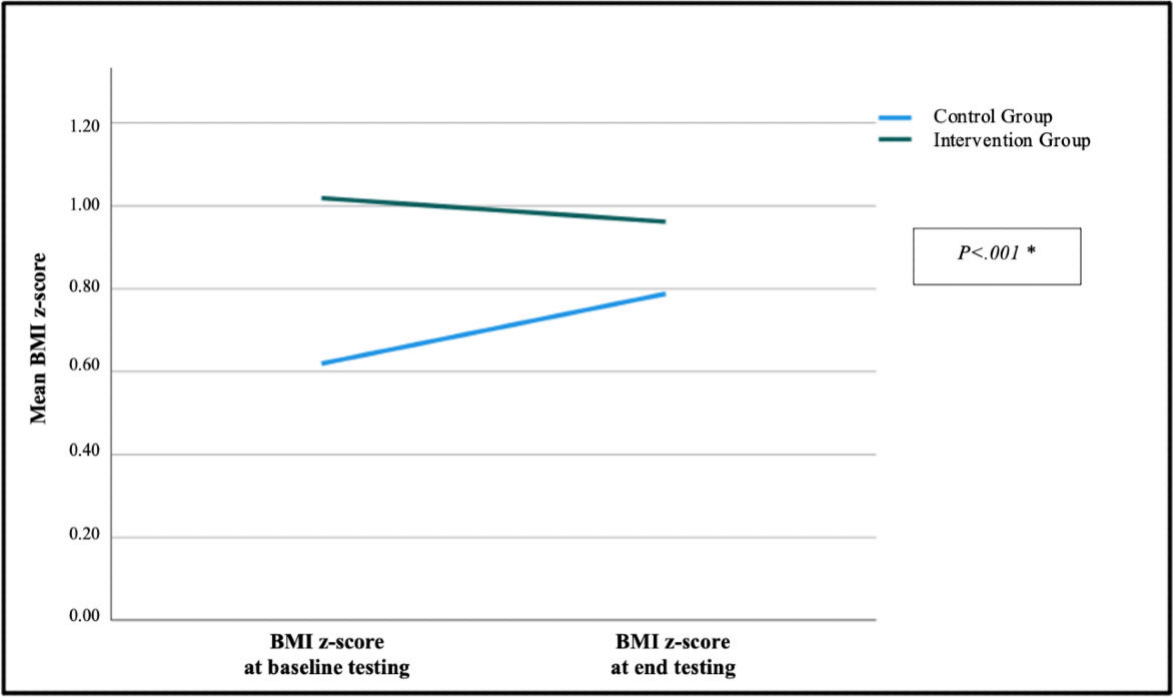
Change in BMI z-score during study period. *Mann–Whitney *U* test: *P*<.001.

After the implementation of the web-based study, the average BMI z-score decreased by −0.06 (SD 0.21) in the intervention group and increased by +0.17 (SD 0.34) in the control group; this was statistically significant (*P*<.001) (Figure 4).

Within the normal-weight BMI category at baseline, there was a statistically significant difference (*P*=.006) between the control group and intervention group for change in BMI z-score. Within the overweight (*P*=.039) and obese (*P*=.062) BMI categories at baseline, there was no statistically significant difference between the control group and intervention group for change in BMI z-score. In the intervention group, the percentage of participants in the overweight BMI category decreased from 22% (n=9) to 17.1% (n=7), representing a change of −4.95%, and the percentage in the obese BMI category increased from 19.5% (n=8) to 22%(n=9; +2.5%). In the control group, the percentage in the overweight BMI category decreased from 25.9%(n=7) to 14.8% (n=4; −11.8%), and the percentage in the obese BMI category increased from 14.8% (n=4) to 22.2% (n=6; +7.4%).

**Figure 5.**
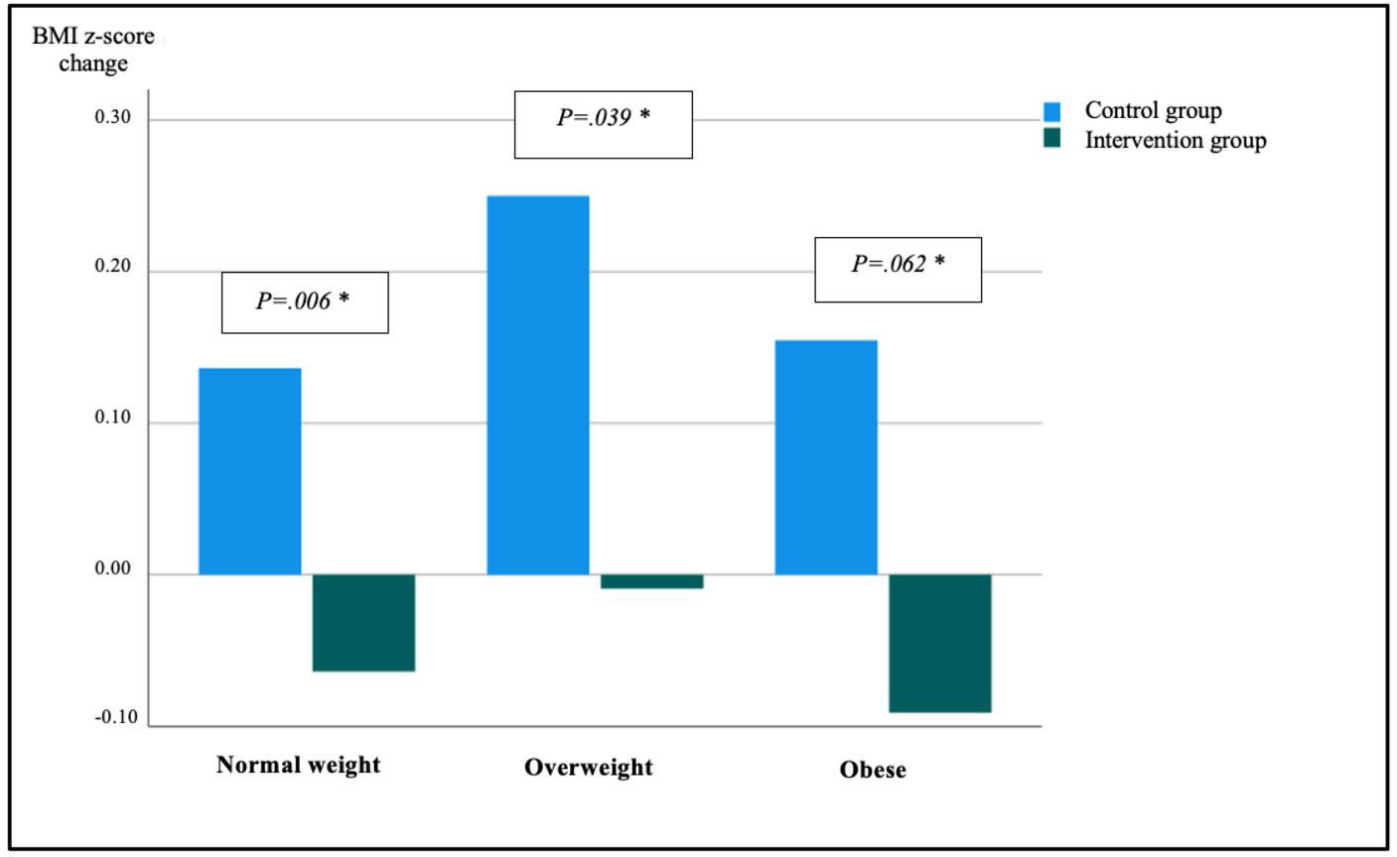
Change in BMI z-score after 15 weeks within the BMI classifications. *Mann–Whitney *U* test for each category: normal weight: *P*=.006; overweight: *P*=.039; obese: *P*=.062. Statistical significance was set after Bonferroni correction at *P*=.017.

Figure 5 shows the exact change for BMI z-score within the BMI classification at baseline.

### Change in BMI z-Score After Intervention According to Sex

**Figure 6.**
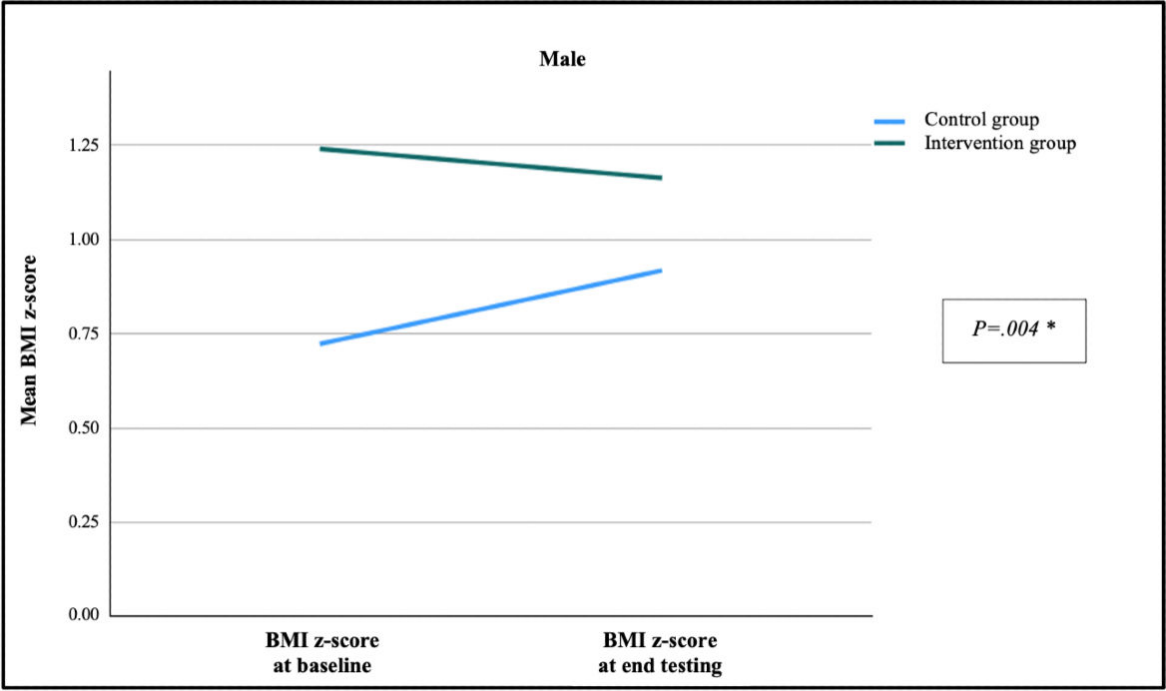
Changes in BMI z-score after 15 weeks in boys according to study group. *Mann–Whitney *U* test: *P*=.004.

Over the study period, the average BMI z-score for girls in the intervention group decreased by −0.04 (SD 0.23) and increased by +0.14 (SD 0.43) in the control group (*P=*.01). Among the boys, BMI z-score decreased by −0.08 (SD 0.19) in the intervention group and increased by +0.19 (SD 0.27) in the control group (*P*=.004). Figures6  7 show the change for BMI z-score over the study period by sex and study group at baseline.

**Figure 7.**
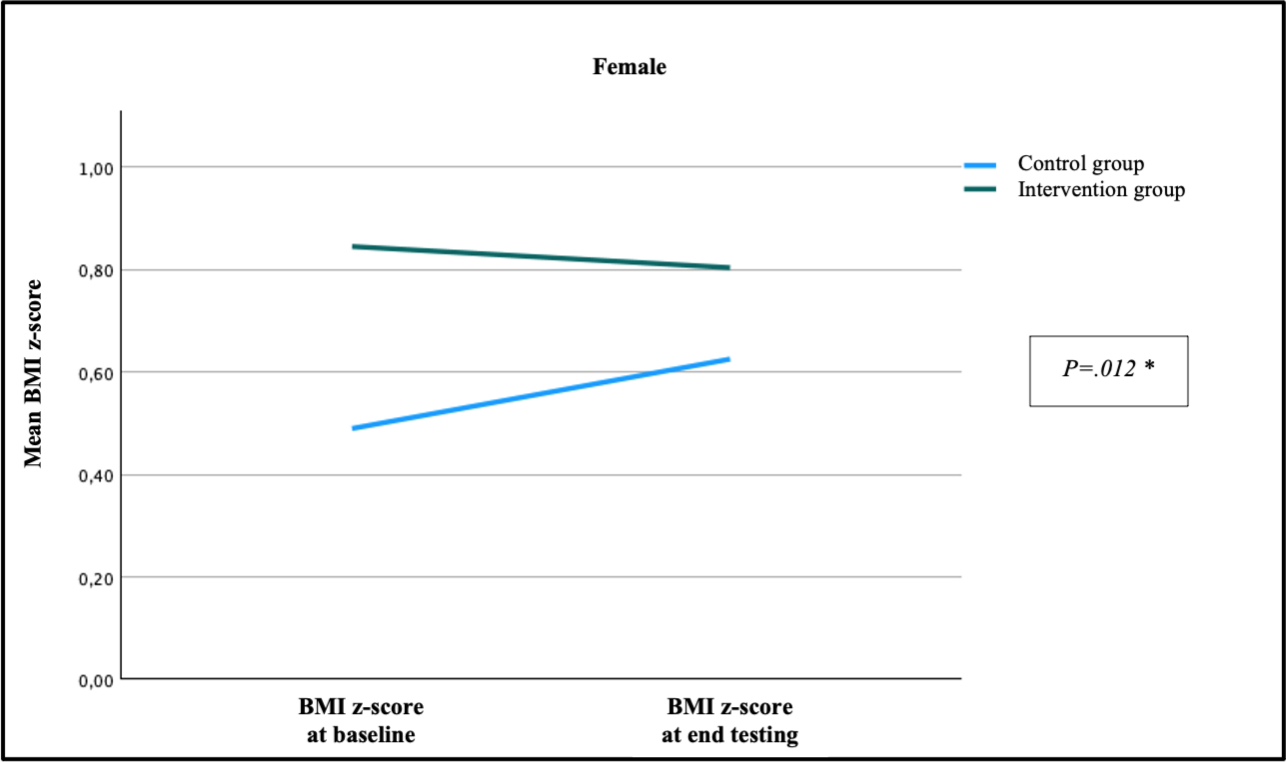
Changes in BMI z-score after 15 weeks in girls according to study group. *Mann–Whitney *U* test: *P*=.01.

## Discussion

In Austria, EDDY is one of the few science-based projects for the prevention of childhood overweight and obesity and its secondary causes. When creating the EDDY online program, we aimed to demonstrate the effect of a web-based nutritional education and PA training course with email support on BMI z-score in primary school children during the COVID-19 pandemic. A 15-week nutritional education and PA intervention was performed, and a control group of the same age and at the school, but from the prior school year, was used for analysis. The mean BMI z-score in the intervention group decreased significantly, by 0.06, over the study period. Here, it should be noted that the control group was measured during the first period of the pandemic, which might have affected the results due to different life circumstances. Recent studies from Austria show that the first period of the pandemic had the biggest impact on children’s BMI [6,36]. However, Brown et al [37] recently also showed a mean decrease of 0.05 in the BMI z-scores of children aged 6 to 12 years in a systematic review of 20 RCTs, which supports the results of this analysis. However, all studies included took place outside of the context of a pandemic. To our knowledge, there is no other comparable online nutritional and PA program that was carried out during the COVID-19 pandemic. Even outside the pandemic context, online interventions as a method of overweight and obesity prevention have not been widely used. Data from international web-based studies are limited. A positive effect on the eating habits of children and adolescents is often reported [38]. The implementation of an online intervention study, such as the EDDY online program, has numerous advantages. A web-based model, which was used for this intervention, can be used with minimal staffing costs [39]. The COVID-19 pandemic led to difficulties in performing ordinary on-site intervention studies, but led to new ways of teaching, such as distance learning. Thus, the study participants in the EDDY online program were able to take part in the intervention despite school closures and social distancing, which could be beneficial in other exceptional situations, like other pandemics. Moreover, by delivering the intervention content through videos, children with language difficulties had the opportunity to repeat the videos as many times as required and choose their own pace. These could be advantages of an online setting, but further studies are required. As reported by the headmaster, the percentage of children in this primary school with a migratory background and a non-German first language was very high. Studies from Austria indicate that children with non-German first languages have a higher prevalence of obesity [40]. This could be supported by the higher mean BMI (intervention group: 21.0 kg/m^2^; control group: 19.4 kg/m^2^) in this study group. The latest Childhood Obesity Surveillance Initiative data indicate a mean BMI of 17.2 kg/m^2^ in urban children aged 7 to 11 years [41]. This could have led to selection bias; therefore, studies need to be carried out at different schools in different regions with a higher number of study participants.

Moreover, using BMI alone to assess children’s body fat has several limitations [42,43]. BMI cannot determine body fat, which is essential to assess cardiovascular and metabolic risk [44]. Adding waist circumference would be a valid way to assess visceral fat [43]. However, recent studies have shown that assessing body composition is the most precise way to determine children’s body fat [42,45] and therefore their obesity-related health level. Due to the COVID-19 pandemic and its mitigation measures in Austria, the determination of body composition was not possible in this study, but further studies should use body composition to evaluate childhood interventions.

Furthermore, studies show that the BMI of children has a high correlation with the BMI of their mothers and fathers [46]. Therefore, the inclusion of parents is essential [47,48]. During the EDDY online program, parents were included in 2 main ways: indirectly, by having the chance to join their children’s intervention (eg, engaging in PA training together), and directly, by receiving newsletters (eg, healthy meal ideas and outdoor PA ideas). Studies show that parents are interested in online interventions, especially if they have high flexibility [49] and if they use a realistic approach [19]. This study provided flexibility for parents and children, as there was no time limit on accessing the newsletters and children could make their own time schedule within a school week. Some studies have also assessed improved effects when children and parents receive separate interventions [50]. Hence, developing a more enhanced and more extensive intervention (eg, with informative videos and online workshops) solely for parents could have potential.

Additionally, web-based studies make it difficult to analyze participation rates and the duration of attendance [21,51]. As the EDDY team used YouTube as a sharing platform due to easy accessibility [21], it was possible to track the views on videos. As it was also possible to watch videos more than once, to engage with the video together with classmates, or to watch the video but not engage in PA, it was not possible to analyze the exact participation rate or duration of attendance. Also, assessing if and how long parents read the newsletters was not possible. Nonetheless, the teachers reported a high participation rate in the beginning, which slightly decreased over the 15-week intervention. Further studies need to develop tools for children and parents to better analyze the duration of attendance and participation rate per study participant. Furthermore, the content delivered in this web-based intervention for children needs to be evaluated through further studies. Borra et al [19] showed that children request “cool” music. Hence, the EDDY team used popular music (eg, Disney music) for PA content. Children also liked the idea of a website solely for them with chatrooms to communicate with other participants or counsellors [19]. In Austria, schools often use online platforms to communicate with children and parents, check attendance rates, and upload school documents. Using a preexisting school platform and creating an exclusive section for children where intervention content can be delivered could, therefore, have potential. This could lead to easier access and handling. Delivering content via online games could also be beneficial [19,52]. Mack et al [53] concluded that video games as an additional tool could help increase the compliance of study participants. Protecting children is very important for parents [19]; data security needs to be considered carefully when using other technologies.

This study has some limitations that need to be addressed and could have influenced the results. First, the number of participating children was small; therefore, our findings may not generalize to Austria’s child population. Our analyses of sex and BMI category should be interpreted with caution due to the even smaller sample sizes. Second, participating children were from a single school that was already familiar with the EDDY program, which could have led to higher feasibility and a better response from the participants and teachers. Third, the number of participants in the intervention and control groups differed, which may have led to limited comparability. Due to the pandemic, many children were missing one measurement point in the control group because of quarantine, sickness, or deregistration from school. Consequently, the results need to be interpreted carefully because of possible selection bias, though the age difference between the 2 study groups was tested statistically prior to the intervention. Fourth, due to mitigation restrictions, the baseline measurements of the intervention group could not be obtained by the EDDY team and were instead obtained by the teachers under close guidance using the school doctor’s equipment, which may have led to minor differences in measuring. Fifth, the observation period differed by 10 weeks between the control and intervention groups, and measurements in the control and intervention groups were also obtained in 2 different years. This might also have impacted the results due to possible differences in lifestyle, environment, or mitigation measures. However, a different approach was not feasible in this study.

Due to the COVID-19 pandemic and restricted physical presence at school, health-prevention interventions were not realizable on site, but technology-based interventions could be used to promote age-appropriate health education. The EDDY online nutritional and PA intervention study with email support aligns with the goal of using a multicomponent approach to tackling childhood obesity and preventing excessive weight gain during periods of social distancing. The study demonstrated a significant decrease in the BMI z-scores of a sample of Viennese primary school children, both in the group overall and when they were divided by sex, after an intervention duration of 15 weeks. Furthermore, the study showed a statistically significant change in the children with normal weight at baseline in the intervention group. However, as this study has several limitations, the results need to be interpreted cautiously. Further studies with fewer limitations are needed to determine whether the results generalize to the entire child population.

This study indicates that this could be a new method to improve children’s health during a pandemic. In the future, assessing body composition, enhancing interventions for parents, evaluating the intervention content, and, in particular, creating methods for the analysis of participation rate and duration of attendance are highly recommended.

## Abbreviations

EDDY: Effect of Sports and Diet Trainings to Prevent Obesity and Secondary Diseases and to Influence Young Children’s Lifestyle
PA: physical activity
VS: Volksschule (German for primary school)
